# Carotid endarterectomy and carotid artery stenting utilization trends over time

**DOI:** 10.1186/1471-2377-12-17

**Published:** 2012-03-29

**Authors:** Matthew R Skerritt, Robert C Block, Thomas A Pearson, Kate C Young

**Affiliations:** 1 Department of Community and Preventive Medicine, Rochester, NY, USA; 2 Clinical and Translational Science Institute, Rochester, NY, USA; 3 Department of Neurology University of Rochester Medical Center, Rochester, NY, USA

## Abstract

**Background:**

Carotid endarterectomy (CEA) has been the standard in atherosclerotic stroke prevention for over 2 decades. More recently, carotid artery stenting (CAS) has emerged as a less invasive alternative for revascularization. The purpose of this study was to investigate whether an increase in stenting parallels a decrease in endarterectomy, if there are specific patient factors that influence one intervention over the other, and how these factors may have changed over time.

**Methods:**

Using a nationally representative sample of US hospital discharge records, data on CEA and CAS procedures performed from 1998 to 2008 were obtained. In total, 253,651 cases of CEA and CAS were investigated for trends in utilization over time. The specific data elements of age, gender, payer source, and race were analyzed for change over the study period, and their association with type of intervention was examined by multiple logistic regression analysis.

**Results:**

Rates of intervention decreased from 1998 to 2008 (P < 0.0001). Throughout the study period, endarterectomy was the much more widely employed procedure. Its use displayed a significant downward trend (P < 0.0001), with the lowest rates of intervention occurring in 2007. In contrast, carotid artery stenting displayed a significant increase in use over the study period (P < 0.0001), with the highest intervention rates occurring in 2006. Among the specific patient factors analyzed that may have altered utilization of CEA and CAS over time, the proportion of white patients who received intervention decreased significantly (P < 0.0001). In multivariate modeling, increased age, male gender, white race, and earlier in the study period were significant positive predictors of CEA use.

**Conclusions:**

Rates of carotid revascularization have decreased over time, although this has been the result of a reduction in CEA despite an overall increase in CAS. Among the specific patient factors analyzed, age, gender, race, and time were significantly associated with the utilization of these two interventions.

## Background

Stroke is the fourth leading cause of mortality in the United States, accounting for over 130,000 deaths each year [1]. More than 85% of all strokes are ischemic in origin [2], with approximately 20% of those attributable to stenosis of the carotid artery [3]. Interventions aimed at primary or secondary stroke prevention in patients with carotid stenosis have evolved over time. Throughout the 1990s, several randomized clinical trials among symptomatic patients (defined as transient or permanent focal neurological deficits) found that carotid endarterectomy (CEA) was superior to, at the time, best medical management [4,5]. This benefit was most significant in patients with greater than 70% stenosis. Similar results were noted among asymptomatic patients. In a sample of 444 men from the VA medical system, Hobson et al. [6] noted a significant reduction in adverse events in the surgery arm of the trial as compared to the medical management arm. Larger and more inclusive studies have reiterated these findings [7,8]. However, CEA is an invasive surgical procedure, and carries the associated risks of such.

Toward the later 1990s, carotid artery stenting (CAS) emerged as a less invasive procedure for revascularization. Despite this advantage, it was unclear whether CAS conferred therapeutic benefit equal to that of CEA with respect to stroke prevention. Several clinical trials have attempted to address this question, with somewhat conflicting results. In studies among symptomatic patients, both the EVA-3 S and SPACE trials failed to show non-inferiority of CAS over CEA in reducing the number of endpoint events [9,10]. Other studies involving both symptomatic and asymptomatic patients found that CAS was either not inferior to [11] or not significantly better than [12] CEA. However, in contrast to SPACE and the early phase of EVA-3 S, CAS was performed with embolic protection in the majority of subjects in both of these studies.

With respect to procedural risk, in 2010, the International Carotid Stenting Study (ICSS) reported that the risk of stroke, death, or myocardial infarction (MI) was significantly higher in the group assigned to stenting [13]. It should be noted that follow-up was for 120 days and embolic protection was not mandated in the study. A later meta-analysis of EVA-3 S, SPACE, and ICSS data found that estimated risk with CAS was twice that of CEA in patients greater than age 70 [14]. Similarly, the risk for stroke, MI, or death with CAS significantly increased with age [15]. There was no age-associated increase noted for CEA. There is also evidence from the CREST trial that women may be at higher risk with CAS as compared to CEA [16].

It now seems clear that the relative benefits of an intervention must be assessed within the context of the individual patient in which it is to be implemented. Patients under the age of 70 or who were excluded from CEA due to high surgical risk were found to have superior outcomes with CAS over CEA or medical management [12]. However, CEA appears to provide the greater benefit to older patients with symptomatic disease [13]. While evidence-based referral of one intervention over the other is presently ambiguous, CAS should be offered as an alternative to surgery for qualified candidates [17]. The number of those qualified, however, is currently limited by a restriction on CAS reimbursement by the Centers for Medicare and Medicaid Services (CMS), an important primary payer of the procedure. At present, stenting (with embolic protection) is reimbursed by the CMS only for those patients excluded from CEA due to high surgical risk and who have ≥ 70% symptomatic stenosis, or who have symptomatic stenosis ≥ 50% or asymptomatic stenosis ≥ 80% and who are enrolled in an FDA-approved clinical trial [18].

In an effort to describe CEA and CAS utilization over time, this study examined the respective prevalence of these interventions in the Nationwide Inpatient Sample (NIS) annually from 1998 to 2008. The NIS is a nationally-representative database of US hospital discharges, and provides comprehensive information across a range of data variables. It was the aim of this study to report on current CEA and CAS utilization trends with respect to several specific patient demographic factors that may influence intervention, and how these factors may have changed over time. The guiding hypothesis of the study was that CAS use has increased over the study period, and that this increase parallels a decline in the rates of CEA.

## Methods

### Database and case identification

The Nationwide Inpatient Sample (NIS), part of the Healthcare Cost and Utilization Project (HCUP), Agency for Healthcare Research and Quality [19], was analyzed annually from 1998 through 2008 This database contains no direct patient identifiers and so is consistent with the definition of "limited data set" under the HIPAA Privacy Rule. For 2008, the 20% stratified sample of the NIS included over eight million discharge records among 1052 participating hospitals in 42 states. Population estimates were generated by incorporating provided discharge weights. From this database, records with a procedure code for carotid endarterectomy were directly identified by the by ICD-9-CM procedure code 38.12, which was used throughout the study period. For years 2004 through 2008, carotid artery stenting cases were identified by the ICD-9-CM procedure code 00.63. As this procedure code was not included in ICD-9-CM coding or the NIS before October 1, 2004, a more indirect approach was required to capture CAS cases prior to this time. To do so, the algorithm of Goodney et al. [20] was employed. Briefly, all records with a procedure code for placement of a non-coronary artery stent (39.90 for years 1998 through 2001, 39.90 and 00.55 for years 2002 through 2008) were obtained. These records were then limited to those in which there was also an ICD-9-CM diagnosis code for cerebrovascular disease (362.30, 362.32, 362.33, 362.34, 362.36, 362.84, 362.89, 433.1, 433.10, 433.11, 433.3, 433.30, 433.31, 433.9, 433.90, 433.91, 435.9, 437.9, and 447.1). In order to exclude those records in which a peripheral stent was placed in a vessel other than the carotid artery, cases with a diagnostic code for peripheral vascular disease were removed (440.2, 440.20, 440.21, 440. 22, 440.23, 440.24, 440.29, 443.8, 443.81, 443.82, 443.89, and 443.9). Also, any record with missing information for age, gender, payer source, and race were excluded. For age, only patients between the ages of 18 and 99 were included, with patients age 90 to 99 grouped to age 90 for analysis. This study was approved by the University of Rochester Medical Center Research Subjects Review Board (RSRB 00036477)

### Statistical analysis

After isolating CEA and CAS cases for each year of the study period, data sets were concatenated in order to permit analysis of trends over time. To determine if the specific patient characteristics of age, gender, payer source, and race changed significantly from 1998 to 2008, univariate models were constructed. In these models, year was treated as the independent variable and its significance represented a significant change in the dependent variable over time. Age was treated as a continuous variable and analyzed using linear regression, while gender (male = 0), payer source (Medicare = 1), and race (white = 1) were dichotomized and analyzed using logistic regression. In the multiple logistic regression models, CEA or CAS (whether or not the discharge record contained a procedure code for CEA or CAS) was treated as the dependent variable, and age, gender, time and categorical (non-dichotomized) payer source and race as the independent variables. Statistical tests were two-sided, with P-values less than 0.05 considered significant. All analyses were performed with SAS 9.2 (SAS Institute, Cary, CA).

## Results

### Trends in CEA and CAS utilization

From 1998 to 2008, 253,651 discharge records with a procedure code for either CEA or CAS were obtained. There was an average of 300 CEA and CAS procedures performed per 100,000 discharges per year. The number of procedures performed per year decreased significantly (P < 0.0001) over the study period, from a high of 358 in 1998 to a low of 262 in 2007 (Table 1).

**Table 1:** Total number of discharges, number of CEA and CAS records, and procedures per 100,000 discharges

Year	Discharges	Combined CEA and CAS records	CEA and CAS Procedures per 100,000 Discharges
1998	6,827,350	24,421	358

1999	7,198,929	24,252	337

2000	7,450,992	24,646	331

2001	7,452,727	24,716	332

2002	7,853,982	23,047	293

2003	7,977,728	23,011	288

2004	8,004,572	21,413	268

2005	7,995,048	21,224	265

2006	8,074,825	22,555	279

2007	8,043,415	21,040	262

2008	8,158,381	23,326	286

	**N = 85,037,949**	**N = 253,651**	**Mean = 300**

Nearly 92% (230,586) of all procedures were for endarterectomies. There was a statistically significant reduction (P < 0.0001) in the number of CEA procedures performed over time, with the lowest rate occurring in 2007. The remaining 8% (23,065) of records coded directly or indirectly for carotid artery stenting. There was an overall increase in the use of stenting over the study period, from a low of 9 procedures per 100,000 discharges in 1998, to a high of 56 per 100,000 discharges in 2006. This trend was statistically significant (P < 0.0001). Table 2 lists the number of records with a procedure code for CEA, the number of combined (indirectly- and directly-coded) CAS cases, and the number of these records per 100,000 discharges for each year of the study period.

**Table 2:** Number of CEA records, combined CAS records, and number of procedures per 100,000 discharges

Year	Discharges	CEA Records	CEA Procedures per 100,000 Discharges	Combined CAS Records	CAS Procedures per 100,000 Discharges
1998	6,827,350	23,782	348	639	9

1999	7,198,929	23,214	322	1,038	14

2000	7,450,992	23,612	317	1,034	14

2001	7,452,727	23,294	313	1,422	19

2002	7,853,982	21,604	275	1,443	18

2003	7,977,728	21,346	268	1,665	21

2004	8,004,572	19,442	243	1,971	25

2005	7,995,048	18,419	230	2,805	35

2006	8,074,825	18,069	224	4,486	56

2007	8,043,415	17,944	223	3,096	38

2008	8,158,381	19,860	243	3,466	42

**Total**	**N = 85,037,949**	**N = 230,586**	**Mean = 273**	**N = 23065**	**Mean = 27**

**Figure 1:**
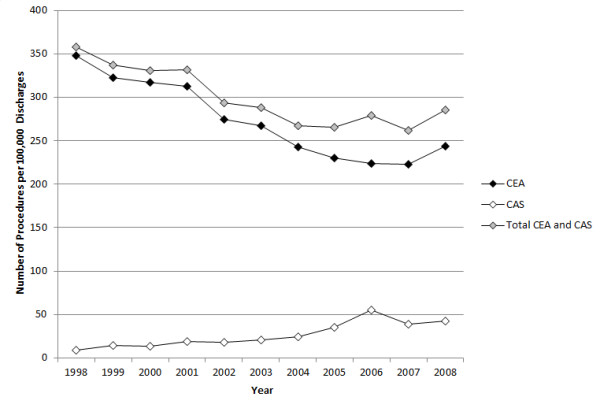
**Trends in CEA and CAS utilization from 1998-2008**.

The number of CEA, CAS, and total CEA and CAS procedures performed from 1998 to 2008 is presented graphically in Figure 1.

### Trends in patient-specific factors

The reduction in CEA and increase in CAS rates noted above may have been influenced by a change in specific patient factors over time. To investigate these changes in those who received either procedure, mean age, the percentage of male patients, patients with Medicare listed as the primary payer, and patients identifying as white were examined for each year of the study period. Univariate logistic regression models were used to test for a change in each of these factors over time. Among the models tested, only race changed significantly over the study period (P < 0.0001). The percentage of patients identifying as white who received either CEA or CAS decreased from a high of 92.87% in 1998, to a low of 87.28% in 2008. This information is presented in Table 3.

**Table 3:** Results of univariate logistic regression analysis testing for a change in each indicated variable over time

Year	1998	1999	2000	2001	2002	2003	2004	2005	2006	2007	2008	1998-2008		
**N**	24,421	24,252	24,646	24,716	23,047	23,011	21,413	21,224	22,555	21,040	23,326	**Slope/OR**	**CI**	**P-Value**

**Age, y** **(SE)**	70.97 (0.06)	70.95 (0.06)	71.11 (0.06)	71.25 (0.06)	71.25 (0.06)	71.04 (0.06)	70.9 (0.07)	71.03 (0.07)	71.12 (0.06)	71.16 (0.07)	71.06 (0.06)	0.005	-0.007-0.0016	0.41

**% Male**	57.26	57.64	57.29	57.3	56.92	56.8	57.05	57.42	57.39	57.32	57.72	1.001	0.998-1.003	0.63

**% Medicare**	71.72	71.73	73.1	73.19	74.68	74.95	73.07	73.7	73.92	73.43	70.98	1.002	1.000-1.005	0.08

**% White**	92.87	91.92	91.47	89.96	89.82	88.56	89.32	89.98	89.48	87.52	87.28	0.949	0.945-0.953	< 0.0001

### Influences on CEA and CAS utilization over Time

To examine the possible confounding effect of changes in the patient demographic factors noted above on CEA and CAS utilization, multiple logistic regression was performed. In the multivariate models, primary payer source and race were treated as categorical variables. Age, gender, race and time were found to significantly predict carotid endarterectomy use. Among these elements, female gender, non-white race, and time predicted lower CEA use. The results of this model are presented in Table 4. For CAS, age, gender, race, and year were again found to contribute significantly to the prediction of intervention, however, their influence was opposite that found for CEA. Female gender, non-white race, and time all positively predicted stenting.

**Table 4:** Results of multivariate logistic regression analysis with CEA as the dependent variable and white race as the reference demographic

Data Element	OR	CI	P-Value
**Age**	1.018	1.016-1.020	< 0.0001

**Gender**	0.908	0.883-0.934	< 0.0001

**Primary Payer Source**	1.004	0.987-1.020	0.6617

**Race: Black**	0.633	0.594-0.674	< 0.0001

**Race: Hispanic**	0.772	0.723-0.825	< 0.0001

**Race: Asian/Pacific Islander**	0.623	0.550-0.706	< 0.0001

**Race: Native American**	0.617	0.513-0.724	< 0.0001

**Race: Other**	0.788	0.716-0.867	< 0.0001

**Year**	0.826	0.822-0.830	< 0.0001

## Discussion and conclusion

In this report it was found that rates of overall carotid revascularization have decreased from 1998 to 2008 in a nationally-representative sample of US hospital discharge records. This reduction was noted even as the median age of US adults increased over the study period [21]. The decrease in revascularization was primarily the result of a reduction in the number of carotid endarterectomy procedures, despite a realized increase in carotid artery stenting. Between 1998 and 2008, the rate of CEA decreased by 36%, while that of CAS increased by 5%. These results are in agreement with, and an extension of, previous reports investigating utilization trends of these interventions [20,22,23].

At present, there is lack of clear clinical directive on which intervention may be superior for primary or secondary stroke prevention. As reported here, carotid endarterectomy has been, and likely will continue to be, the much more widely utilized procedure. From 1998 to 2008, CEA use has decreased by about one third, yet the overall rate of procedures during that time exceeds that of CAS by an order of magnitude. If patients most at risk for procedural complications avoid the surgery, the 5-year risk of stroke or death may be reduced 5 to 6% as compared to medical management alone [7]. However, two caveats must be addressed. First, absolute risk reduction is much more nuanced than the quoted 5 to 6% when patient-specific factors and longer follow-up times are taken into account [24]. Second, medical management of stroke has been refined since early CEA trials comparing the two approaches, and today nearly all patients at risk for stroke are prescribed antihypertensive, antiplatelet, and lipid-lowering therapies. Such an aggressive regimen may be effectively reducing stroke risk and the subsequent need for surgery, particularly in asymptomatic patients [25].

In contrast, rates of carotid artery stenting were found to increase significantly over the study period. The highest rates of intervention were noted in 2006, with a 25% decrease from 2006 to 2008. Interestingly, nearly the lowest rates of CEA occurred in 2006. In 2004, the results of the SAPPHIRE trial were published, indicating that CAS with embolic protection was not inferior to CEA for the prevention of stroke in select patients [11]. That same year the first FDA-approved carotid artery stent was introduced, and, in early 2005, the CMS expanded its reimbursement policies for CAS [18]. Investigation of a correlation between these events is beyond the scope of this work, but it is an intriguing prospect. It remains to be seen if the overall increase in CAS reported here will continue going forward. The recently-published CREST [12] and ICSS [13] trials failed to provide consensus on which procedure conferred the greater benefit, and so future studies in this area will be welcomed. Of interest is the SPACE2 trial, which will compare the effectiveness of best medical management vs. CEA vs. CAS [26].

A primary aim of this study was to identify specific patient demographic factors that may have changed over time, and to examine their potential influence on procedure rates. Among those factors analyzed, there was a significant decrease in the percentage of patients identifying as white who received intervention. This decrease remained significant for total CEA and CAS rates, as well as for CEA (P < 0.0001) and CAS (P = 0.02) independently. However, whites were still the overwhelming recipients of carotid revascularization. Although there is an overall higher number of whites in the general population [21], part of the explanation may also be that white patients are more commonly affected by atherosclerotic carotid artery disease than non-whites [27]. Within the context of endarterectomy specifically, several studies have provided some understanding into why minority patients may be less likely to have surgery. Black patients were found to have higher rates of complicating comorbid conditions [28] and faced increased barriers to quality care [29]. In addition, there are racial differences in the decision to have surgery, with blacks significantly more averse than whites to CEA [30]. Despite these findings, it has been reported that when clinically indicated and adjusting for ancillary factors, any difference in the delivery of CEA between white and non-white patients is attenuated [31].

When the additional factors of age, gender, payer source, and time were included in multivariate models, it was found that age, gender, race, and time were significant predictors of either CEA or CAS. Increased age, male gender, white race, and earlier in the study period were significant positive predictors of CEA use. Payer source did not reach statistical significance in either model, nor did it change significantly over time in the univariate model. As noted in Table 3, Medicare was listed as the primary payer in 73% of cases throughout the study period. Despite the current reimbursement limitation for carotid artery stenting by the CMS, rates continued to increase over the study period. This may suggest that, other factors notwithstanding, CAS delivery may accelerate should this limitation be lifted in the future.

Several limitations to this study should be noted. First, as described elsewhere, the algorithm used to capture carotid artery stenting prior to 2004 suffers from a lack of certainty in the identification of "true" CAS cases [20]. However, any bias introduced as a result would be systematic, and tend not to influence the change in rates of CAS over time. Also, the data used in this report was taken from a de-identified, discharge-based database. Therefore, the denominators used to calculate intervention rates likely represent several non-unique patients, which may artificially lower the numbers presented here. Finally, this work is a comment on the change over time of CEA and CAS, and as such, does not provide the ability to explain unambiguously why these changes have occurred. Importantly, the third side to the stroke prevention triad, medical management, was excluded from analysis here. How the revision in medical therapy for stroke and stroke prevention has contributed to the change in rates in this study population is unknown.

In conclusion, it was found that overall rates of carotid revascularization have decreased from 1998-2008 in a nationally-representative sample of US hospital discharges. This decrease was primarily the result of a reduction in the number of carotid endarterectomy procedures, despite an increase in the rate of carotid artery stenting. Among the patient-specific factors analyzed, race changed significantly over time, and age, gender, race, and time significantly predict utilization of intervention. Several recent reports investigating the utility of CEA as compared to CAS for the prevention of stroke have been published. However, future work remains to adequately inform the deployment of these interventions to the patients for whom the greatest benefit will be conferred.

## Competing interests

The authors declare that they have no competing interests.

## Authors' contributions

MRS designed and carried out the study, analyzed and interpreted the data, wrote, revised, and gave final approval of the article, and was overall responsible for the study and its publication. RCB critically revised and gave final approval to the article. TAP critically revised and gave final approval to the article and obtained funding for support of this work. KCY designed the study, and revised and gave final approval of the article. All authors read and approved the final manuscript.

## Sources of funding

This work was supported by an NIH T32 Institutional Training Grant (HL007937) to the University of Rochester Medical Center, Clinical and Translational Science Institute.

## Pre-publication history

The pre-publication history for this paper can be accessed here:

http://www.biomedcentral.com/1471-2377/12/17/prepub
